# Application of Artificial Intelligence on Psychological Interventions and Diagnosis: An Overview

**DOI:** 10.3389/fpsyt.2022.811665

**Published:** 2022-03-17

**Authors:** Sijia Zhou, Jingping Zhao, Lulu Zhang

**Affiliations:** ^1^Department of Psychiatry, Guangzhou First People's Hospital, The Second Affiliated Hospital of South China University of Technology, Guangzhou, China; ^2^Mental Health Institute of the Second Xiangya Hospital, Central South University, Changsha, China; ^3^Chinese National Clinical Research Center on Mental Disorders, Changsha, China; ^4^Department of Psychiatry, Chinese National Technology Institute on Mental Disorders, Changsha, China; ^5^Hunan Key Laboratory of Psychiatry and Mental Health, Changsha, China

**Keywords:** artificial intelligence, deep learning, machine, intervention, diagnosis

## Abstract

**Background:**

Innovative technologies, such as machine learning, big data, and artificial intelligence (AI) are approaches adopted for personalized medicine, and psychological interventions and diagnosis are facing huge paradigm shifts. In this literature review, we aim to highlight potential applications of AI on psychological interventions and diagnosis.

**Methods:**

This literature review manifest studies that discuss how innovative technology as deep learning (DL) and AI is affecting psychological assessment and psychotherapy, we performed a search on PUBMED, and Web of Science using the terms “psychological interventions,” “diagnosis on mental health disorders,” “artificial intelligence,” and “deep learning.” Only studies considering patients' datasets are considered.

**Results:**

Nine studies met the inclusion criteria. Beneficial effects on clinical symptoms or prediction were shown in these studies, but future study is needed to determine the long-term effects.

**Limitations:**

The major limitation for the current study is the small sample size, and lies in the lack of long-term follow-up-controlled studies for a certain symptom.

**Conclusions:**

AI such as DL applications showed promising results on clinical practice, which could lead to profound impact on personalized medicine for mental health conditions. Future studies can improve furthermore by increasing sample sizes and focusing on ethical approvals and adherence for online-therapy.

## Introduction

Increasing prevalent mental problems including depressive disorders, anxiety and related disorders are affecting the population's health conditions in a negative way. Frequency of mental health and artificial intelligence (AI) publications are expanding dramatically over the past few years (1). Deep learning (DL) methods would be important to handle complicated medical data-sets, building vigorous data-sets to share between institutions. Recent AI studies suggested promising directions for employing technology including deep learning in improving our understanding of mental health diagnosis and treatment (2, 3). Robotic interactions assist in providing the availability of information regarding individual emotional changes and keep daily records on cognitive changes (4). Technology such as Machine Learning has been implemented to solve mental health problems; emerging data in this field have provided insights into application of AI technologies in psychological treatments (5). Research regarding deep learning and its application in the medical ground is clinically increasingly relevant to therapeutic applications in treating mental disorders, which leads the aim of this review.

The innovation of machine learning endorses a data-driven approach in which a general-purpose program automatically learns elements from data (6), with no prior expertise needed (7). Methods developed based on deep learning in the machine learning field have effectively improved the current state of psychotherapy, and applied in a wide range of applications, such as identifying objects in images (8), recognizing speech (9), translating languages (10), understanding the genetic determinants of diseases (11), and predicting health status using electronic health records (12). With current technology covering online-treatments and robotic care for dementia and autism disorder (13, 14), AI applications in mental health can bring insights into new treatment approaches, Hilty et al. (15) provided evidence that AI brings opportunities to involve rural areas that have insufficient medical resources, better patient response, and save time for clinicians in the U.S. Additionally, DL is utilized in interventions for schizophrenia and is suggested to be effective in reducing anxiety by helping patients to detect emotional changes and thought patterns and to enhance flexible thinking styles. Fiske et al. (16) offer evidence that DL algorithms such as Tess, specifically designed for practical and accessible use of internet psychotherapy. Even though integrative psychological AI is not designed to replace the role of a trained therapist, it emerges as a feasible option for providing support for patients in need. People designed Tess to flag expressions that indicate emotional distress; the study found that it helped reduce depression and anxiety symptoms among users. Tess interprets psychological terms such as cognitive distortions and provides detailed advice for ascertaining and tackling difficult situations, which can be employed in areas that have fewer clinical resources. Nonetheless, a recent review by Horn and Weisz (17) argued that most psychological treatments focus on the aspects of cognition, behavior and emotion of a person much more than the biological features. These client features, which may vary adequately between individuals, apart from the interactions between these features and the environment, would need to be improved by AI to make it as precise and as useful as AI is for medical and pharmaceutical intervention. The objective of the review is to highlight potential applications of AI on psychological interventions and diagnosis, along with searching for new treatments, or complementary ways to improve effects of scale assessment or drug treatment.

Deep learning is a category of machine learning allows advanced problem-solving techniques of complicated non-linear pattern recognition of spatial samples. The proposition is to employ neural networks (NN) to learn a stratified representation of the data and continuously produce interpretable outcomes (18). Diagnosis for mental health disorders such as depression includes obtaining and extracting relevant data from DL databases to analyze patients who display classic symptoms of depression, such as feelings of sadness, emptiness, loss of interest or pleasure, and sleep disturbances. Recent studies (19, 20) reviewed the literature applying Deep Learning-based methods to analyze neuroimaging and EEG data for psychiatric disorders (including ASD, SZ, ADHD), DL technology would be a promising tool for the diagnosis and classification of patients with mental health disorders.

## Methods

For this mini-review, articles published in English from January 2010 to December 2020 were searched in Pubmed (https://www.ncbi.nlm.nih.gov/pubmed/) and Web of Science using the terms “psychological interventions,” “diagnosis on mental health disorders,” “artificial intelligence,” and “deep learning.” We have based the criteria for selection on various components, the benefit of this approach is that we could narrow down a more precise range of articles. Publications were only included in the analysis if patients' datasets contained clinical, genetic, or medical information. Nine studies, which have been summarized in Table 1, met these inclusion criteria. We included studies showing positive results to discuss the constructive influences of different AI applications when implemented in psychological diagnosis and interventions.

**Table 1:** Summary of studies of artificial intelligence technology in psychological interventions and diagnosis.

**Authors**	**Objective**	**Form of delivery**	**Sample size**	**Conclusions**
Fitzpatrick et al. (21)	The efficacy of self-help program Woebot for college students who self-identify as having symptoms of anxiety and depression	Woebot	70 University students	Woebot is accessible, engaging, and effective way to deliver CBT.
Mohr et al. (7)	Uncovering the potential of mobile health and its effect on mental health research and treatment	Personal sensing methods including smart phones, wearable, social media and computers	Layered, hierarchical model for translating raw sensor data into markers of behaviors and states related to mental health	Integration into Existing Models of Care, Behavioral Intervention Technologies
Kuang and He (22)	Prediction of ADHD status	fMRI	449 Subjects	DL used for discrimination of ADHD with fMRI data.
Geraci et al. (23)	Prediction of youth depression	DFNN	366 Patients	Individuals who meet the inclusion–exclusion criteria for depression research were detected.
Navarro-Haro et al. (24)	The benefits of VR Dialectical Behavioral Therapy (DBT) mindfulness skills training technique	Virtual Reality (VR) and DBT	44 participants	Participants reported less sadness, anger, and anxiety after VR
Gosling et al. (25)	Internet delivery of fully automated self-help CBT-I designed to reduce insomnia and prevent depression	Online CBT-I	1,600 community-dwelling adults	Program showed significant results
Spence et al. (26)	The study examined the relative efficacy of online (NET) vs. clinic (CLIN) delivery of cognitive behavior therapy (CBT) in the treatment of anxiety disorders in adolescents	Online CBT	15 clinically anxious adolescents aged 12 to 18	Minimal differences between NET and CLIN, both showed effectiveness
Ewbank et al. (27)	Quantifying and measuring the delivery of psychotherapy using deep learning	Internet-enabled CBT for the treatment of a mental health disorder from 2012 to 2018	17,572 patients (90,934 therapy session transcripts)	Deep learning model applied to the large-scale clinical data set of CBT session transcripts and generate a quantifiable measure
Castro et al. (28)	Evaluate the effectiveness of telephone-administered psychotherapy for depression in adults	A meta-analysis	Studies that included randomized controlled trials (RCTs) examining the impact of telephone-administered psychotherapy on depressive symptoms.	Telephone-administered psychotherapy showed beneficial effects on depression severity when compared to control conditions

AI is the theory and development of computer systems able to perform tasks that usually require human intelligence, including components such as visual perception, speech recognition, and decision-making. Machine Learning is a type of artificial intelligence that includes systems that can learn from data, identify patterns, and make decisions with minimal human intervention, ML techniques include support vector machines, decision trees, neural networks, latent Dirichlet allocation, and clustering. ML focus on building systems that can automatically improve through experience using advanced statistical and probabilistic techniques (29). The application of ML in mental health has demonstrated a wide range of advantages across the fields of diagnosis, interventions and clinical administration (30). Researchers have applied ML to social media and online community information to decide the individual and linguistic and psychological features most predictive for successful alcohol abstinence (31). Deep Learning is a type of machine learning method based on learning data representations. Gkotsis et al. (32) revealed that Natural language processing of electronic health records in social media can be used to analyse people's mental health status on a daily basis. By implementing a deep learning approach, Gkotsis et al. (32) automatically recognized source of “in the moment” posts on social media platforms and classify posts related to mental illness following psychological disorder themes.

## Results

### Identification and Diagnosis

Deep learning (DL) has emerged as a way to examine large data-sets to reinforce risk detection (33). DL can screen through large data-sets including clinical files and social media information to recognize factors that might increase the risk of depression, including certain personality traits, such as low self-esteem or being too dependent, self-critical or pessimistic, or some traumatic or stressful events, or relatives with a history of mental disorders and so on.

Data collection and recognition allows clinical institutions to prevent suicidal attempts, and the analysis of prediction help to identify individuals in crisis so that we can intervene with emotional support, psycho-educational resources, and alerts for the need for emergency assistance (34). At the population level, algorithms can reduce suicidal attempt risks by identifying groups that are at risk or suicide hotspots, which help resource mobilization, policy reform or advocate local resources to help out and inform. The application of advanced machine learning technology to analyze the relations between different risk factors can improve early stages of mental health disorders and help early intervention and detection of patients with a tendency of getting ill ahead of time.

Zheng et al. (35) provides strong evidence of the connection between mental illness and attempted suicidal behavior. They conclude that the risk of suicide attempts among the people with one mental disorder at least is more than 10 times greater than those without. DL technologies are capable of detecting and intervening population who has the predisposition of attempting suicide, for example, Annette (36) highlighted salient risk factors like previous suicidal behavior. The diathesis-stress model of predicting population who are more vulnerable to mental health illnesses is being taken into consideration (37). The potential benefits that DL approaches can provide include precise risk hierarchy, informing specialists of the demand for mental health intervention, as a result, more integrated care plans can be developed. The method is likely to allow facilitation of the effectiveness of interventions to mitigate risk through targeting for providers in actual time. Therefore, developing and advancing personalized health care plans by analyzing available data from clinical institutions, record the intervention progress can help reducing suicidal risks.

Brain neural network plays an important role in the neuropathological mechanism of depression. DL learning about neuro-imaging (fMRI scans) helps to detect brain changes and subsequent mental disorders (38). Moreover, DL can learn features from the raw data without the need for a prior feature selection, resulting in a process that is more objective and less bias-prone. Kang et al. (39) demonstrated how integrative DL applications are used to differentiate panic disorder from other anxiety disorders using HRV (heart rate variability), thus using objective measurements to check biological differences and brain chemistry adjustment can be meaningful in the detection of mental health disorders, with DL technologies, identifications and detection could be more comprehensive in various ways.

Diagnosis for mental health disorders such as depression includes obtaining and extracting relevant data from DL databases to analyze patients who display classic symptoms of depression, such as feelings of sadness, emptiness, loss of interest or pleasure, and sleep disturbances. Online evaluation can help the specialist to quickly assess psychological state of patients, as mobile devices are more available nowadays and people can go through a psychological assessment at their convenience. Kalmady et al. (40) classified patients with schizophrenia using fMRI scans, best algorithm was Ensemble model: accuracy = 87%.

### Psychological Interventions

Depressed mood and loss of interest or pleasure in daily activities are the main characteristics of depression (41). The impact of depression can be extensive and disrupt social, occupational, and academic functioning. In China, it is estimated that about 100 million people suffer from various mental disorders, and 16 million are severely affected by their conditions. Research showed that Asians, especially those of Chinese descent are less likely to report their negative mood than individuals from Western cultures (42, 43). Not willing to approach for help could lead to a lower reporting rate for depression.

Psychotherapy generally termed as treating depression by talking about your condition and related problems with a therapist. Psychotherapeutic interventions are also known as talk therapy. Applying deep learning to large clinical data sets can provide significant insights into psychotherapy, informing people of the result of new treatments and helping standardize clinical practice and shed light upon interventions for relapse prevention (44). Pastor et al. (45) indicated that digital tools such as robotic partners or mobile devices can remind elderly people to take steps to reduce stress, reach out to family and friends, and getting long-term maintenance treatment. Nguyen et al. (46) used deep learning predictor with 2 hidden layers in DNN to identify whether patients with depression would respond favorably to bupropion. Squarcina et al. (47) suggested that patients' response to treatment vary across studies, might be because of inconsistent methodologies. Hence, deep learning algorithms could stimulate data-driven autoencoder to produce outcome effectively. Moreover, DL distinguishes between psychological interventions and brings out the best available treatment option.

Conversational technology has been increasingly integrated into the delivery of psychological interventions such as social therapy (48), cognitive behavioral therapy (21) and trauma therapy (49). Since the program responds to the presented dialogue, intervention delivery can be tailored to a patient's emotional state and clinical needs (48). Similar technologies, although preliminary, are being incorporated into smart phones to allow voice assistants to recognize and respond to user mental health concerns (50).

Internet-enabled CBT is effective for the treatment of depression clinically, and so is widely adopted in nowadays (27). Horn and Weisz (17) indicated that is not yet feasible to expect AI applications to be as contributive in psychotherapy compared to the extent in medical field, considering the striking difference between treatment response of psychotherapy and pharmacology. Unlike quantifiable and measurable treatments in medical practice (such as the dosage of prescribed drugs), the method of psychological interventions is sessions of confidential discussions between the patient and the clinician. Therapy can be in the form of online sessions, videos or workbooks accessible through internet descriptions and programs can be guided by a mental health professional independently. Ewbank et al. (27) illustrates the clinical relevance of supplementary online therapy; however, whether or not deep learning algorithms could be a substitute for a human therapist is unknown.

RCTs and reviews reported beneficial effects of exercise in moderately-depressed patients (51). Data from electronic health records can show early life adversity, stressful life events, and very proximal factors such as lifestyle, nutrition, and sport. Computers and Internet-based programs have great potential to produce more cost-effective psychological assessment and treatment. Taylor and Luce (2) stated that computer-assisted therapy appears to be as effective as a face-to-face treatment in treating anxiety disorders and depression, Internet support groups also may be effective and have advantages over face-to-face therapy. The results suggest that online assessment could reduce therapist contact time without adjusting or accommodating schedules. VR is a useful apparatus to improve exposure therapy, Botella et al. (52) demonstrated that virtual reality exposure therapy's (VRET) is effective for phobias. Parsons and Rizzo (53) showed large declines in anxiety symptoms following VRET. AI technology has also shown to be effective in improving the social skills of children with autism spectrum disorder (54). This means that children with ASD can practice, imitate and cooperate with their robotic partners that they have a greater chance of improving their interactions with adults. Such automated interaction aims to help children with ASD in need of acquiring proper skills such as social, self-reflection and management, with the aim of the children being able to apply the skills learned with the robot peer to their relationships with human peers.

## Discussion

AI technology holds great promises to transform mental health care and is not without potential pitfalls. Deep learning technology and online-interventions have significant meaning to mental health domain in that patients are given limited time during actual psychotherapy, compared to therapy hours being extended and accumulated through online-courses. Ewbank et al. (27) suggested that a therapist may accumulate abundant experience during his/her career which represents valuable expertise in CBT. Deep learning might allow us to make use of this knowledge to provide benefits to online interventions, and help improve the efficacy of psychotherapy. Shatte et al. (30) summarized the methods and applications of ML in mental health and found that four main application domains emerged: detection and diagnosis, prognosis treatment and support, public health, and research and clinical administration. DL applications can also be relevant in treatment studies in which new modes of intervention delivery methods can be explored. Durstewitz et al. (55) reviewed deep learning applications in psychiatry and suggested that they may provide insights similar to machine learning in psychiatry.

Other ethical considerations include severe consequences for online counseling (56). Particularly, online counseling and DL was noted as inappropriate for suicidal and psychotic clients, though online operations offer clinicians the chance to help people that were previously not able to be engaged in psychotherapy (57). Limitations are highlighted as studies point out contraindications for online therapy, noting that “severe pathology and risky behavior such as lethally suicidal conditions may not be appropriate for online work”. There are other limitations of online counseling, one of which is the inability to read non-verbal cues. The visual anonymity of much online counseling is often perceived as a strength; however, since body language plays a crucial role in face-to-face therapy, its absence online is likely to also bring challenges to practitioners and clients (58–60). Despite the reality that online counseling is developing very rapidly, there's very limited research focused on the phenomena, although more and more literature has emerged around online communication in general. Adherence to conversational agents is another factor to be considered when utilizing AI in psychotherapy (21). Taking findings from this more general research (61–64), however, problematic because counseling has many unique characteristics as a form of communication. In light of the prominence of evidence-based practice in the counseling industry, research specifically addressing the problems of online counseling practice is essential to access and flexibility as the strongest perceived advantages, identified by 85% of practitioners as strengths of online mental health services.

## Conclusion

Overall, AI such as DL applications can be useful to treat mental disorders at the client's convenience. However, the field of AI and psychological interventions still have to be explored, and further research is needed to address the broader ethical and societal concerns of these technologies to negotiate the best research.

## Author Contributions

SZ, LZ, and JZ developed the framework for the mini-review. SZ and LZ wrote the manuscript and approved its submission. LZ edited the manuscript. All authors contributed to the article and approved the submitted version.

## Funding

This study was supported by grants from the Science and Technology Program of Guangzhou (Grant No. 202002030262) and the Natural Science Foundation of Guangdong Province (Grant No. 2017A030313809).

## Conflict of Interest

The authors declare that the research was conducted in the absence of any commercial or financial relationships that could be construed as a potential conflict of interest.

## Publisher's Note

All claims expressed in this article are solely those of the authors and do not necessarily represent those of their affiliated organizations, or those of the publisher, the editors and the reviewers. Any product that may be evaluated in this article, or claim that may be made by its manufacturer, is not guaranteed or endorsed by the publisher.
